# Supplementary material for: Outcomes of minimal change disease without nephrotic range proteinuria
Source: PLoS One. 2023 Aug 17;18(8):e0289870. doi: 10.1371/journal.pone.0289870 (PMC10434851; doi:10.1371/journal.pone.0289870)
Supplement: S1 Table — Creatinine before biopsy: The lowest value of serum creatinine 6months before renal biopsy, DM: Diabetes mellitus, CHD: Coronary heart disease, SBP: Systolic blood pressure, DBP: Diastolic blood pressure, Cr: Creatinine, GFR: Estimated glomerular filtration rate by CKD-EPI equation, AKI: Acute kidney injury based on the lowest creatinine value during the follow-up period, Max UPCR before biopsy: The highest value of UPCR during the 6 months before biopsy, UPCR: Spot urine protein to creatinine ratio (g/g cr). (DOCX) [file pone.0289870.s001.docx]

**S1 Table. Characteristics of patients with minimal change disease according to the highest amount of proteinuria during 6 months before renal biopsy**

| **Characteristic** | **Completeness** | **Non-NS2** | **NS2** | **p-value** |
| --- | --- | --- | --- | --- |
|  | **of data (%)** | **(n=16)** | **(n=63)** |  |
| **Age (years)** | 100.0 | 52.4 ± 15.9 | 54.0 ± 20.0 | 0.729 |
| **Male (n, %)** | 100.0 | 7 (43.8) | 31 (49.2) | 0.696 |
| **DM (n, %)** | 100.0 | 5 (31.3) | 7 (11.1) | 0.045 |
| **Hypertension (n, %)** | 100.0 | 7 (43.8) | 35 (55.6) | 0.398 |
| **History of CHD (n, %)** | 100.0 | 0 (0.0%) | 4 (6.3%) | 0.301 |
| **Weight (kg)** | 100.0 | 63.9 ± 11.6 | 65.4 ± 12.8 | 0.644 |
| **SBP (mmHg)** | 100.0 | 118 ± 15 | 125 ± 18 | 0.109 |
| **DBP (mmHg)** | 100.0 | 70 ± 10 | 73 ± 12 | 0.232 |
| **Cholesterol (mg/dl)** | 93.7 | 255 ± 158 | 367 ± 115 | 0.016 |
| **Glucose (mg/dl)** | 100.0 | 129 ± 34 | 109 ± 29 | 0.046 |
| **Protein (g/dl)** | 92.4 | 5.8 ± 1.2 | 4.4 ± 0.8 | <0.001 |
| **Albumin (g/dl)** | 92.4 | 3.3 ± 1.0 | 2.2 ± 0.6 | <0.001 |
| **Hemoglobin (g/dl)** | 100.0 | 13.4 ± 1.9 | 13.5 ± 2.1 | 0.896 |
| **Creatinine (mg/dl)** | 100.0 | 0.97 ± 0.90 | 1.19 ± 0.80 | 0.368 |
| **Creatinine before biopsy (mg/dl)** | 100.0 | 0.68 ± 0.22 | 0.66 ± 0.24 | 0.728 |
| **GFR (ml/min/1.73 m2)** | 100.0 | 96 ± 29 | 77 ± 35 | 0.041 |
| **AKI at biopsy (n, %)** | 100.0 | 1 (6.3) | 35 (55.6) | <0.001 |
| **Stage 1 (n, %)** | 100.0 | 0 (0.0) | 14 (40.0) | 0.005 |
| **Stage 2 (n, %)** | 100.0 | 0 (0.0) | 9 (25.7) |  |
| **Stage 3 (n, %)** | 100.0 | 1 (100.0) | 12 (34.2) |  |
| **AKI requiring dialysis (n, %)** | 100.0 | 0 (0.0) | 5 (7.9) | 0.244 |
| **Max. UPCR before biopsy (g/g cr)** | 100.0 | 1.80 ± 0.87 | 10.88 ± 6.19 | 0.009 |
| **<0.30 g/g cr (n, %)** | 100.0 | 0 (0.0) | 0 (0.0) | <0.001 |
| **0.30-2.99 g/g cr (n, %)** | 100.0 | 16 (100.0) | 0 (0.0) |  |
| **>3.00 g/g cr (n, %)** | 100.0 | 0 (0.0) | 63 (100.0) |  |
| **UPCR at biopsy (g/g cr)** | 100.0 | 1.58 ± 0.98 | 9.55 ± 6.46 | <0.001 |
| **<0.30 g/g cr (n, %)** | 100.0 | 2 (12.5) | 1 (1.6) | <0.001 |
| **0.30-2.99 g/g cr (n, %)** | 100.0 | 14 (87.5) | 3 (4.8) |  |
| **>3.00 g/g cr (n, %)** | 100.0 | 0 (0.0) | 59 (93.6) |  |
