# Supplementary material for: Outcomes of minimal change disease without nephrotic range proteinuria
Source: PLoS One. 2023 Aug 17;18(8):e0289870. doi: 10.1371/journal.pone.0289870 (PMC10434851; doi:10.1371/journal.pone.0289870)
Supplement: S2 Table — LM findings: Light microscopy findings, Mes: Mesangial, IF findings: Immunofluorescent microscopy findings, EM findings: Electron microscopy findings P-values determined using the Mann-Whitney test, uc: Uncountable. (DOCX) [file pone.0289870.s002.docx]

**S2 Table.** **Renal pathologic findings of patients according to the amount of proteinuria**

| **Characteristic** | **Completeness** | **Non-NS** | **NS** | ***p-value*** |
| --- | --- | --- | --- | --- |
|  | **of data (%)** | **(n=20)** | **(n=59)** |  |
| **LM findings** |  |  |  |  |
| **Glomeruli** |  |  |  |  |
| **Number of glomeruli (n)** | 100.0 | 33.7 ± 20.3 | 35.7 ± 16.7 | 0.364 |
| **Global sclerosis (%)** | 100.0 | 7.5 ± 10.9 | 9.3 ± 11.2 | 0.505 |
| **Segmental sclerosis (%)** | 100.0 | 0.0 ± 0.0 | 0.0 ± 0.0 | 1.000 |
| **Crescent (%)** | 100.0 | 0.0 ± 0.0 | 0.0 ± 0.0 | 1.000 |
| **Ischemic change (%)** | 100.0 | 0.46 ± 2.03 | 0.08 ± 0.46 | 0.721 |
| **Increase of mes. matrix (n, %)** | 100.0 | 2 (10.0) | 10 (16.9) | 0.720 |
| **Tubulointerstitium** |  |  |  |  |
| **Tubular atrophy (n, %)** | 100.0 | 12 (60.0) | 49 (83.1) | 0.061 |
| **Interstitial fibrosis (n, %)** | 100.0 | 11 (55.0) | 47 (79.7) | 0.042 |
| **Interstitial inflammation (n, %)** | 100.0 | 8 (40.0) | 42 (71.2) | 0.017 |
| **Vessel** |  |  |  |  |
| **Arteriosclerosis (n, %)** | 100.0 | 0 (0.0) | 4 (6.8) | 0.567 |
| **Intimal thickening (n, %)** | 100.0 | 6 (30.0) | 26 (44.1) | 0.304 |
| **IF staining of glomeruli (intensity)** |  |  |  |  |
| **IgG** | 100.0 | 0.15 ± 0.37 | 0.09 ± 0.24 | 0.871 |
| **IgM** | 100.0 | 0.38 ± 0.43 | 0.31 ± 0.42 | 0.464 |
| **IgA** | 100.0 | 0.20 ± 0.38 | 0.10 ± 0.24 | 0.342 |
| **C3** | 100.0 | 0.13 ± 0.22 | 0.06 ± 0.19 | 0.110 |
| **C1q** | 100.0 | 0.18 ± 0.29 | 0.16 ± 0.29 | 0.818 |
| **Fibrinogen** | 100.0 | 0.00 ± 0.00 | 0.01 ± 0.07 | 0.560 |
| **Kappa chain** | 100.0 | 0.03 ± 0.11 | 0.01 ± 0.07 | 0.419 |
| **Lambda chain** | 100.0 | 0.10 ± 0.21 | 0.04 ± 0.17 | 0.101 |
| **IF staining of glomeruli (n ≥1+, %)** |  |  |  |  |
| **IgG** | 100.0 | 3 (15.0) | 2 (3.4) | 0.100 |
| **IgM** | 100.0 | 5 (25.0) | 13 (22.0) | 0.766 |
| **IgA** | 100.0 | 3 (15.0) | 2 (3.4) | 0.100 |
| **C3** | 100.0 | 0 (0.0) | 1 (1.7) | 1.000 |
| **C1q** | 100.0 | 1 (5.0) | 3 (5.1) | 1.000 |
| **Fibrinogen** | 100.0 | 0 (0.0) | 0 (0.0) | uc |
| **Kappa chain** | 100.0 | 0 (0.0) | 0 (0.0) | uc |
| **Lambda chain** | 100.0 | 0 (0.0) | 1 (1.7) | 1.000 |
| **EM findings** |  |  |  |  |
| **Electron dense deposits** | 100.0 |  |  |  |
| **Mesangium (n, %)** | 100.0 | 0 (0.0) | 0 (0.0) | uc |
| **Subepithelial space (n, %)** | 100.0 | 0 (0.0) | 0 (0.0) | uc |
| **Subendothelial space (n, %)** | 100.0 | 0 (0.0) | 0 (0.0) | uc |
| **Podocyte foot process** |  |  |  |  |
| **Diffuse effacement (n, %)** | 100.0 | 8 (40.0) | 55 (93.2) | <0.001 |
