# Supplementary material for: Outcomes of minimal change disease without nephrotic range proteinuria
Source: PLoS One. 2023 Aug 17;18(8):e0289870. doi: 10.1371/journal.pone.0289870 (PMC10434851; doi:10.1371/journal.pone.0289870)
Supplement: S3 Table — For the amount of proteinuria, a multiple linear regression model that adjusted for age, sex, blood pressures, and pathologic findings of podocyte effacement (none, focal, and diffuse) was used. For the presence of NS, a multiple logistic regression model that adjusted for age, sex, serum glucose, pathologic findings of interstitial fibrosis, interstitial inflammation, tubular atrophy, and podocyte effacement (none, focal, and diffuse) was used. NS: Nephrotic-range proteinuria, CI: Confidence interval, RR, relative risk. (DOCX) [file pone.0289870.s003.docx]

**S3 Table.** **Effect of podocyte effacement severity on proteinuria**

|  |  | B | 95% CI of B | | *p-value* |
| --- | --- | --- | --- | --- | --- |
| For amount of proteinuria* | Podocyte effacement | 4.061 | 1.441 | 6.681 | 0.003 |
|  |  | RR | 95% CI of RR | | *p-value* |
| For presence of NS* | Podocyte effacement |  |  |  | <0.001 |
|  | Focal | 2.394 | 0.405 | 14.149 | 0.335 |
|  | Diffuse | 11.728 | 2.364 | 58.185 | 0.003 |
