# Supplementary material for: Outcomes of minimal change disease without nephrotic range proteinuria
Source: PLoS One. 2023 Aug 17;18(8):e0289870. doi: 10.1371/journal.pone.0289870 (PMC10434851; doi:10.1371/journal.pone.0289870)
Supplement: S4 Table — Min UPCR after biopsy: The lowest value of UPCR during the follow-up period starting 1 month after biopsy, Max UPCR after biopsy: The highest value of UPCR during the follow-up period starting 1 month after biopsy, FU duration after biopsy: Follow-up duration between renal biopsy and the last test of UPCR, RAS: Renin-angiotensin-system, CR: Complete remission of proteinuria <0.3 g/g creatinine, relapse: UPCR >3.0 g/g creatinine after achieving CR of UPCR, SD: Presence of steroid dependency in a patient where relapse of UPCR occurred during steroid tapering or within 2 weeks after cessation of steroids, renal event: Any decrease of GFR of more than 50% during a follow-up visit compared to that at renal biopsy, GFR <15 ml/min/1.73 m2, or development of ESRD, The first CR (n, %) among patients with UPCR >3.00 g/g cr: In the Non-NS group, patients who had increased proteinuria of >3.00 g/g cr during observation period. The first relapse among patients with UPCR <0.30 g/g cr: In the Non-NS group, patients who had UPCR of <0.30 g/g cr at any time of period. (DOCX) [file pone.0289870.s004.docx]

**S4 Table. Outcomes of minimal change disease according to the highest amount of proteinuria during 6 months before renal biopsy**

| **Outcomes** | **Completeness** | **Non-NS2** | **NS2** | **p-value** |
| --- | --- | --- | --- | --- |
|  | **of data (%)** | **(n=16)** | **(n=63)** |  |
| **FU duration after biopsy (months)** | 100.0 | 60.4 ± 56.1 | 72.9 ± 48.2 | 0.424 |
| **Number of laboratory tests during follow-up period (/month)** | 100.0 | 0.42 ± 0.25 | 0.70 ± 0.79 | 0.021 |
| **Min. UPCR after biopsy (g/g cr)** | 100.0 | 0.21 ± 0.25 | 0.13 ± 0.26 | 0.279 |
| **<0.30 g/g cr (n, %)** | 100.0 | 12 (75.0) | 59 (93.7) | 0.027 |
| **0.30-2.99 g/g cr (n, %)** | 100.0 | 4 (25.0) | 4 (6.3) |  |
| **>3.00 g/g cr (n, %)** | 100.0 | 0 (0.0) | 0 (0.0) |  |
| **Max. UPCR after biopsy (g/g cr)** | 100.0 | 3.00 ± 4.10 | 8.01 ± 9.48 | 0.043 |
| **<0.30 g/g cr (n, %)** | 100.0 | 3 (18.8) | 11 (17.5) | 0.122 |
| **0.30-2.99 g/g cr (n, %)** | 100.0 | 8 (50.0) | 16 (25.4) |  |
| **>3.00 g/g cr (n, %)** | 100.0 | 5 (31.3) | 36 (57.1) |  |
| **UPCR at last visit (g/g cr)** | 100.0 | 0.91 ± 1.74 | 1.27 ± 4.47 | 0.614 |
| **<0.30 g/g cr (n, %)** | 100.0 | 10 (62.5) | 48 (76.2) | 0.279 |
| **0.30-2.99 g/g cr (n, %)** | 100.0 | 5 (31.3) | 9 (14.3) |  |
| **>3.00 g/g cr (n, %)** | 100.0 | 1 (6.3) | 6 (9.5) |  |
| **Creatinine at last visit (mg/dl)** | 100.0 | 1.02 ± 0.68 | 1.01 ± 0.65 | 0.954 |
| **GFR at last visit (ml/min/1.73 m2)** | 100.0 | 87 ± 29 | 85 ± 30 | 0.756 |
| **Treatment after renal biopsy** |  |  |  |  |
| **RAS blocker (n, %)** | 100.0 | 10 (62.5) | 33 (52.4) | 0.468 |
| **Anti-hypertensive medication (n, %)** | 100.0 | 11 (68.8) | 43 (68.3) | 0.970 |
| **Anti-diabetic medication (n, %)** | 100.0 | 5 (31.3) | 24 (38.1) | 0.612 |
| **Treatment for induction of the first CR** |  |  |  |  |
| **Immunosuppression (n, %)** | 100.0 | 6 (37.5) | 56 (88.9) | <0.001 |
| **Steroid only (n, %)** | 100.0 | 5 (31.3) | 23 (36.5) |  |
| **Steroid and calcineurin inhibitor (n, %)** | 100.0 | 0 (0.0) | 28 (44.4) |  |
| **Steroid and cyclophosphamide (n, %)** | 100.0 | 1 (6.3) | 5 (7.9) |  |
| **Highest dose of prednisolone (mg/kg/day)** | 100.0 | 0.97 ± 0.05 | 0.88 ± 0.17 | 0.222 |
| **Total dose of prednisolone until CR or 1st relapse (mg)** | 100.0 | 4477 ± 1468 | 7063 ± 3518 | 0.081 |
| **Outcomes** |  |  |  |  |
| **The first CR (n, %) among patients with UPCR >3.00 g/g cr (n, %)** | 100.0 | 12/16 (75.0) | 58/63 (92.1) | 1.000 |
| **The first relapse among patients with UPCR <0.30 g/g cr (n, %)** | 100.0 | 5/12 (41.7) | 28/58 (48.3) | 0.676 |
| **SD at the first relapse (n, %)** | 100.0 | 1/16 (6.3) | 10/63 (15.9) | 0.122 |
| **Number of relapses after biopsy (/year)** | 100.0 | 0.23 ± 0.33 | 0.25 ± 0.41 | 0.830 |
| **CR at last visit (n, %)** | 100.0 | 13 (65.0) | 45 (76.3) | 0.384 |
| **Slope of GFR (ml/min/1.73 m2/year)** | 100.0 | (-)4.67 ± 8.97 | 3.13 ± 12.9 | 0.026 |
| **Renal events during follow-up (n, %)** | 100.0 | 2 (12.5) | 17 (27.0) | 0.226 |
| **ESRD during follow-up (n, %)** | 100.0 | 1 (6.3) | 9 (14.3) | 0.388 |
| **Death during follow-up (n, %)** | 100.0 | 0 (0.0) | 3 (4.8) | 0.374 |
