# Supplementary material for: Outcomes of minimal change disease without nephrotic range proteinuria
Source: PLoS One. 2023 Aug 17;18(8):e0289870. doi: 10.1371/journal.pone.0289870 (PMC10434851; doi:10.1371/journal.pone.0289870)
Supplement: S5 Table — For presence of AKI at diagnosis, a multiple logistic regression model adjusted for age, sex, serum albumin, eGFR, blood pressures, and severity of podocyte effacement was used. For the first remission in patients with UPCR >3.0 g/g cr: A Cox proportional hazards model adjusted for age; sex; and pathologic findings of deposition of IgA, IgG, lambda chains, or interstitial inflammation, was used. For the first relapse in patients with UPCR <0.3 g/g cr: A Cox proportional hazards model adjusted for age, sex, and steroid treatment was used. For the number of relapses in patients with UPCR <0.3 g/g cr: A multiple linear regression model adjusted for age, sex, serum cholesterol, and steroid treatment was used. For renal events, a Cox proportional hazards model adjusted for age, sex, presence of AKI at renal biopsy, presence of hypertension, eGFR, pathologic deposition of IgA, RAS blockade medication, anti-hypertensive drugs, anti-diabetic drugs, immunosuppressive drugs, and number of relapses of nephrotic range proteinuria was used. For the remission of proteinuria at the last visit, a Cox proportional hazards model adjusted for age; sex; serum albumin; presence of diabetes mellitus; first remission of proteinuria; relapse of proteinuria; and pathologic findings of changes in the mesangial matrix, interstitial inflammation, and presence of atherosclerosis, was used. AKI: Acute kidney injury, eGFR: Estimated glomerular filtration rate, cr: Creatinine, RAS: Renin-angiotensin system, UPCR: Urine protein/creatinine ratio, MCD: Minimal change disease. (DOCX) [file pone.0289870.s005.docx]

**S5 Table. Effect of presence of nephrotic range proteinuria at diagnosis of MCD on each event**

| Event | p-value |
| --- | --- |
| Presence of AKI at diagnosis | 0.972 |
| First remission in patients with UPCR >3.0 g/g cr | 0.726 |
| First relapse in patients with UPCR <0.3 g/g cr | 0.107 |
| Number of relapses in patients with UPCR <0.3 g/g cr | 0.553 |
| Renal events | 0.225 |
| Remission of proteinuria at last visit | 0.116 |
