# Supplementary material for: Outcomes of minimal change disease without nephrotic range proteinuria
Source: PLoS One. 2023 Aug 17;18(8):e0289870. doi: 10.1371/journal.pone.0289870 (PMC10434851; doi:10.1371/journal.pone.0289870)
Supplement: Attachment — Submitted filename: REBUTTAL LETTER.docx [file pone.0289870.s007.docx]

**Rebuttal letter**

**Reviewer #1:**

We really appreciate your recommendations.

Answers to your each recommendation are as below.

- Some patients in the NS group received cyclophosphamide- it would be good to discuss how this could have impacted the results.

Thank you for recommendation.

We added sentences as below (12p).

“4 patients were treated with steroid and cyclophosphamide. They all head heavy proteinuria over at least 6 g/g at initial manifestation. Podocyte effacement on kidney pathology was diffused and wide in all 4 patients. Because they all showed severe nephrotic syndrome, they needed intense immunosuppressive therapy. They achieved the first CR without steroid dependency during withdrawal, and AKI, developed in 2 of them, was relieved.” (12p)

- One of the definitions of renal events was decrease in eGFR by 50% at a follow-up visit compared to that at renal biopsy. Perhaps it would have been better to compare to the baseline Cr (or average Cr in weeks/few months prior to kidney biopsy). Because patients could have been pre-renal or have any other cause of AKI that was not really related to MN, and this could have biased the results.

Thank you for kind recommendation.

We had also collected lowest creatinine during 6 months before renal biopsy. Serum creatinine was a little higher in non-NS group than in NS group without statistical significance. However, it did not show difference between two groups in an aspect of clinical outcome with serum creatinine. We added this to result section as below. (16p)

“The response rate to steroid treatment in patients with UPCR ≥3.00 g/g creatinine at any point during follow-up was 100% (8/8 patients) in the Non-NS group and 92.3% (51/55 patients) in the NS group (p=1.000). The frequency of first relapses in patients with UPCR <0.30 g/g creatinine (p=0.782) and the number of relapses (p=0.830) did not differ between the groups. At the final visit, the CR rate was 73.4% (58/79), showing no significant difference between the groups (Fig 2 and Table 3). The days to the first remission were 10.7 ± 16.5 days in non-NS group and 8.7 ± 20.2 days in NS group (p=0.701) (Fig 3-A). Except 9 patients who did not achieve CR, the days to the first relapse was 35.5 ± 49.0 days in non-NS group and 29.6 ± 43.6 days in NS group (p=0.637) (Fig 3-B). The eGFR during the observation period was significantly better in the NS group compared to the Non-NS group because of higher incidence of AKI at renal biopsy in the NS group. The ratio of last serum creatinine by the lowest serum creatinine 6 months before biopsy was 1.47 (± 0.50) in non-NS group, when was 1.59 (± 0.82) in NS group (p=0.213). The delta of last serum creatinine to the lowest serum creatinine 6 months before biopsy was 0.32 (± 0.50) in non-NS group and 0.36 (± 0.60) in NS group, respectively (p = 0.679). Overall, The incidence rate of renal events, ESRD events, or mortality was not different between the groups (Table 3).” (16p)

- It would be great to mention if, among the patients who were on dialysis, how many of them were on dialysis at time of biopsy, since the Creatinine would be falsely low, and thus, biasing the end point "decrease in GFR by 50%).

I appreciate for your comment.

Five patients started hemodialysis due to acute kidney injury when biopsy was done. However, because there were at least more than 3 days between the day of biopsy and the day of starting hemodialysis, all baseline creatinine was measured before starting dialysis.

- Is there a reason why Highest dose of prednisolone was used to compare groups as opposed to average dose of prednisolone?

Thank you for recommendation.

We wanted to emphasize on the similar dose of steroid between two groups. So, we suggested both the highest dose of steroid, mostly the start dose, and the total dose of steroid together. As we know, although there is standard regimen for MCD treatment, low-dose steroid regimen or short-term use of steroid would be possible in some situations. In this study, patients in two groups used similar dose of steroid regardless of initial among of proteinuria, once steroids were used.

- Did any patients have any findings of diabetic nephropathy in addition to the MN findings which could have impacted the results?

I appreciate for your recommendation.

We reviewed our data again in an aspect of diabetic nephropathy. Although there were 15 % of patients (12 patients) who were diagnosed with diabetes mellitus, diabetic nephropathy was exclusive diagnosis. Because we excluded the collection of data who were suspected as diabetic nephropathy clinically or pathologically in methodology.

Reviewer #2.

Thank you for your thoughtful recommendations.

Sentences below are my answers to your comments.

- I would suggest to use a Kaplan Meier curve to present the comparison between groups in regards to time to complete remission after treatment and time to first relapse

We appreciate for your recommendations.

Kaplan-Meier graphs for the 1^st^ remission and 1^st^ relapse was added to the manuscript as below (figure 3-A and 3-B).Because as the mean times were not different between two groups, the description was added as below (16p)

Figure 3. Survival time from the kidney biopsy to the first remission (A) and time from the first remission to the first relapse (B).

(A)

(B)

Linear line indicates the Kaplan-Meier graph line in Non-NS group. Dashed line indicate that in NS group.

“The response rate to steroid treatment in patients with UPCR ≥3.00 g/g creatinine at any point during follow-up was 100% (8/8 patients) in the Non-NS group and 92.3% (51/55 patients) in the NS group (p=1.000). The frequency of first relapses in patients with UPCR <0.30 g/g creatinine (p=0.782) and the number of relapses (p=0.830) did not differ between the groups. At the final visit, the CR rate was 73.4% (58/79), showing no significant difference between the groups (Fig 2 and Table 3). The days to the first remission were 10.7 ± 16.5 days in non-NS group and 8.7 ± 20.2 days in NS group (p=0.701) (Fig 3-A). Except 9 patients who did not achieve CR, the days to the first relapse was 35.5 ± 49.0 days in non-NS group and 29.6 ± 43.6 days in NS group (p=0.637) (Fig 3-B). The eGFR during the observation period was significantly better in the NS group compared to the Non-NS group because of higher incidence of AKI at renal biopsy in the NS group. The ratio of last serum creatinine by the lowest serum creatinine 6 months before biopsy was 1.47 (± 0.50) in non-NS group, when was 1.59 (± 0.82) in NS group (p=0.213). The delta of last serum creatinine to the lowest serum creatinine 6 months before biopsy was 0.32 (± 0.50) in non-NS group and 0.36 (± 0.60) in NS group, respectively (p = 0.679). Overall, The incidence rate of renal events, ESRD events, or mortality was not different between the groups (Table 3).” (16p)

Please find below specific comments in regards to the manuscript content.

Lines 85-88
We defined MCD pathologically as near normal findings on light microscopy, except for mild expansion of the mesangium, and no electron deposit and focal or diffuse podocyte effacement on electron microscopy

Comment
I believe the definition of MCD used could be more descriptive, for example:
“Near normal findings on light microscopy, except for mild expansion of the mesangium or global glomerulosclerosis, which is a nonspecific finding that can also be seen. There is absence of segmental sclerosis. There are no complement or immunoglobulin deposits on immunofluorescence microscopy. On electron microscopy there is diffuse effacement of podocyte foot processes. There is absence of electron dense deposits or thickening of the glomerular basement membrane.
Note. The characteristic histologic lesion in MCD is diffuse effacement (>50-80%) of the epithelial foot processes on electron microscopy. The proposed diagnosis of MCD on this manuscript includes patients with focal or diffuse foot process effacement. Limited foot process effacement (<50%) suggests the process is probably not MCD, unless the patient has been treated with partial response. However, on line 90 it is mentioned that patients who had immunosuppressive treatment before renal biopsy were excluded.
I suggest to incorporate in the definition of MCD the exclusion of segmental sclerosis. Focal foot process effacement is more commonly associated with FSGS.

Thank you for your kind recommendation.

The sentences were corrected as you suggested as below (6p in the manuscript)

“We defined MCD pathologically as near normal findings on light microscopy, except for mild expansion of the mesangium or global glomerulosclerosis which could also be seen as nonspecific findings. Segmental sclerosis was not seen. There were no complement or immunoglobulin deposits on immunofluorescence microscopy. Diffuse effacement of podocyte foot processes was seen on electron microscopy.” (6p)

Lines 129-131
As we had previously described [10], glomerular lesions such as global sclerosis, segmental sclerosis, glomerular ischemic change, and crescentic changes were reported as a proportion of the total number of glomeruli in an evaluated specimen

Comment
I found this statement confusing. On lines 85-88 it is mentioned that MCD was defined as near normal findings on light microscopy. But here it states that segmental sclerosis, glomerular ischemic change, crescentic changes are reported. If these findings are present, it would not be consistent with a diagnosis of MCD.

I appreciate for your comment.

Because we intended to descript the pathological diagnostic steps to diagnose MCD, “renal pathology” section might have mentioned all pathological descriptions in general. To clarify our intend, we corrected sentences as below . And we also abbreviated table 2 and added details of pathologic findings in supplementary table 1.

“As we had previously described [10], pathologic diagnosis contains following contents. Glomerular lesions such as global sclerosis, segmental sclerosis, glomerular ischemic change, and crescentic changes were reported as a proportion of the total number of glomeruli in an evaluated specimen. A semi-quantitative assessment of changes in mesangial cellularity, mesangial matrix, tubular atrophy, and interstitial inflammation and fibrosis was performed, with the results classified as normal, mild, moderate, moderate to severe, and severe. Vascular abnormality was defined as the presence of arteriolar hyalinosis and arteriosclerosis. The immunofluorescence study was performed using the classic direct technique with antibodies against 8 proteins (IgG, IgM, IgA, C3, C1q, fibrinogen, kappa and lambda chains). The results were reported semi-quantitatively as negative (0), trace (0.5) and 1–3 positive (1-3). The findings by electron microscopy were descripted as followings; presence of electron dense deposits in the area of the mesangium, subendothelium, and subepithelium; and severity of foot process effacement of the podocytes, which were reported as none, focal (mild, moderate, moderate to severe, severe), or diffuse.” (8p in the manuscript)

**Table 2. Characteristics of patients with minimal change disease according to the amount of proteinuria**

| **Characteristic** | **Completeness** | **Non-NS** | **NS** | **p-value** |
| --- | --- | --- | --- | --- |
|  | **of data (%)** | **(n=20)** | **(n=59)** |  |
| **Age (years)** | 100.0 | 53.0 ± 15.2 | 53.9 ± 20.5 | 0.550 |
| **Male (n, %)** | 100.0 | 8 (40.0) | 30 (50.8) | 0.447 |
| **DM (n, %)** | 100.0 | 5 (25.0) | 7 (11.9) | 0.168 |
| **Hypertension (n, %)** | 100.0 | 10 (50.0) | 32 (54.2) | 0.799 |
| **History of CHD (n, %)** | 100.0 | 0 (0.0%) | 4 (6.8%) | 0.567 |
| **Weight (kg)** | 100.0 | 61.8 ± 11.4 | 66.2 ± 12.8 | 0.169 |
| **SBP (mmHg)** | 100.0 | 118 ± 15 | 125 ± 18 | 0.118 |
| **DBP (mmHg)** | 100.0 | 69 ± 11 | 73 ± 11 | 0.181 |
| **Cholesterol (mg/dl)** | 93.7 | 262 ± 144 | 372 ± 116 | <0.001 |
| **Glucose (mg/dl)** | 100.0 | 127 ± 33 | 108 ± 29 | 0.012 |
| **Protein (g/dl)** | 92.4 | 5.7 ± 1.2 | 4.3 ± 0.7 | <0.001 |
| **Albumin (g/dl)** | 92.4 | 3.3 ± 1.0 | 2.1 ± 0.5 | <0.001 |
| **Hemoglobin (g/dl)** | 100.0 | 13.4 ± 1.8 | 13.5 ± 2.1 | 0.664 |
| **Creatinine (mg/dl)** | 100.0 | 0.89 ± 0.81 | 1.23 ± 0.81 | 0.008 |
| **Creatinine before biopsy (mg/dl)** | 100.0 | 0.65 ± 0.21 | 0.66 ± 0.24 | 0.871 |
| **GFR (ml/min/1.73 m2)** | 100.0 | 98 ± 27 | 75 ± 36 | 0.012 |
| **AKI at biopsy (n, %)** | 100.0 | 1 (5.0) | 35 (59.3) | <0.001 |
| **Stage 1 (n, %)** | 100.0 | 0 (0.0) | 14 (23.7) | <0.001 |
| **Stage 2 (n, %)** | 100.0 | 0 (0.0) | 9 (15.3) |  |
| **Stage 3 (n, %)** | 100.0 | 1 (5.0) | 12 (20.3) |  |
| **AKI requiring dialysis (n, %)** | 100.0 | 0 (0.0) | 5 (8.3) | 0.322 |
| **Max. UPCR before biopsy (g/g cr)** | 100.0 | 2.63 ± 1.92 | 11.22 ± 6.25 | <0.001 |
| **<0.30 g/g cr (n, %)** | 100.0 | 0 (0.0) | 0 (0.0) | <0.001 |
| **0.30-2.99 g/g cr (n, %)** | 100.0 | 16 (80.0) | 0 (0.0) |  |
| **≥3.00 g/g cr (n, %)** | 100.0 | 4 (20.0) | 59 (100.0) |  |
| **UPCR at biopsy (g/g cr)** | 100.0 | 1.36 ± 1.00 | 10.16 ± 6.21 | <0.001 |
| **<0.30 g/g cr (n, %)** | 100.0 | 3 (15.0) | 0 (0.0) | <0.001 |
| **0.30-2.99 g/g cr (n, %)** | 100.0 | 17 (85.0) | 0 (0.0) |  |
| **≥3.00 g/g cr (n, %)** | 100.0 | 0 (0.0) | 59 (100.0) |  |

**Supplementary Table S1. Renal pathologic findings of patients according to the amount of proteinuria**

| **Characteristic** | **Completeness** | **Non-NS** | **NS** | ***p-value*** |
| --- | --- | --- | --- | --- |
|  | **of data (%)** | **(n=20)** | **(n=59)** |  |
| **LM findings** |  |  |  |  |
| **Glomeruli** |  |  |  |  |
| **Number of glomeruli (n)** | 100.0 | 33.7 ± 20.3 | 35.7 ± 16.7 | 0.364 |
| **Global sclerosis (%)** | 100.0 | 7.5 ± 10.9 | 9.3 ± 11.2 | 0.505 |
| **Segmental sclerosis (%)** | 100.0 | 0.0 ± 0.0 | 0.0 ± 0.0 | 1.000 |
| **Crescent (%)** | 100.0 | 0.0 ± 0.0 | 0.0 ± 0.0 | 1.000 |
| **Ischemic change (%)** | 100.0 | 0.46 ± 2.03 | 0.08 ± 0.46 | 0.721 |
| **Increase of mes. matrix (n, %)** | 100.0 | 2 (10.0) | 10 (16.9) | 0.720 |
| **Tubulointerstitium** |  |  |  |  |
| **Tubular atrophy (n, %)** | 100.0 | 12 (60.0) | 49 (83.1) | 0.061 |
| **Interstitial fibrosis (n, %)** | 100.0 | 11 (55.0) | 47 (79.7) | 0.042 |
| **Interstitial inflammation (n, %)** | 100.0 | 8 (40.0) | 42 (71.2) | 0.017 |
| **Vessel** |  |  |  |  |
| **Arteriosclerosis (n, %)** | 100.0 | 0 (0.0) | 4 (6.8) | 0.567 |
| **Intimal thickening (n, %)** | 100.0 | 6 (30.0) | 26 (44.1) | 0.304 |
| **IF staining of glomeruli (intensity)** |  |  |  |  |
| **IgG** | 100.0 | 0.15 ± 0.37 | 0.09 ± 0.24 | 0.871 |
| **IgM** | 100.0 | 0.38 ± 0.43 | 0.31 ± 0.42 | 0.464 |
| **IgA** | 100.0 | 0.20 ± 0.38 | 0.10 ± 0.24 | 0.342 |
| **C3** | 100.0 | 0.13 ± 0.22 | 0.06 ± 0.19 | 0.110 |
| **C1q** | 100.0 | 0.18 ± 0.29 | 0.16 ± 0.29 | 0.818 |
| **Fibrinogen** | 100.0 | 0.00 ± 0.00 | 0.01 ± 0.07 | 0.560 |
| **Kappa chain** | 100.0 | 0.03 ± 0.11 | 0.01 ± 0.07 | 0.419 |
| **Lambda chain** | 100.0 | 0.10 ± 0.21 | 0.04 ± 0.17 | 0.101 |
| **IF staining of glomeruli (n ≥1+, %)** |  |  |  |  |
| **IgG** | 100.0 | 3 (15.0) | 2 (3.4) | 0.100 |
| **IgM** | 100.0 | 5 (25.0) | 13 (22.0) | 0.766 |
| **IgA** | 100.0 | 3 (15.0) | 2 (3.4) | 0.100 |
| **C3** | 100.0 | 0 (0.0) | 1 (1.7) | 1.000 |
| **C1q** | 100.0 | 1 (5.0) | 3 (5.1) | 1.000 |
| **Fibrinogen** | 100.0 | 0 (0.0) | 0 (0.0) | uc |
| **Kappa chain** | 100.0 | 0 (0.0) | 0 (0.0) | uc |
| **Lambda chain** | 100.0 | 0 (0.0) | 1 (1.7) | 1.000 |
| **EM findings** |  |  |  |  |
| **Electron dense deposits** | 100.0 |  |  |  |
| **Mesangium (n, %)** | 100.0 | 0 (0.0) | 0 (0.0) | uc |
| **Subepithelial space (n, %)** | 100.0 | 0 (0.0) | 0 (0.0) | uc |
| **Subendothelial space (n, %)** | 100.0 | 0 (0.0) | 0 (0.0) | uc |
| **Podocyte foot process** |  |  |  |  |
| **Diffuse effacement (n, %)** | 100.0 | 8 (40.0) | 55 (93.2) | <0.001 |

Lines 138-139
The findings assessed using electron microscopy included presence of electron dense deposits in the area of the mesangium, subendothelium, and subepithelium

Comment
I found this statement confusing. On line 87 you mention that one of the criteria to define MCD was absence of electron dense deposits. But here you mention that you are evaluating electron dense deposits based on location. If electron dense deposits are present this would NOT be consistent with a diagnosis of MCD

I am really appreciate for your comment.

As you said, it should be not descriptive of MCD. We just wanted to descript reviewed pathologic findings in detail. We found that there were no electron deposits as we expected. To clarify our intends, we fixed sentences as below. (8p) And we also abbreviated table 2 and added details of pathologic findings in supplementary table 1.

“As we had previously described [10], pathologic diagnosis contains following contents. Glomerular lesions such as global sclerosis, segmental sclerosis, glomerular ischemic change, and crescentic changes were reported as a proportion of the total number of glomeruli in an evaluated specimen. A semi-quantitative assessment of changes in mesangial cellularity, mesangial matrix, tubular atrophy, and interstitial inflammation and fibrosis was performed, with the results classified as normal, mild, moderate, moderate to severe, and severe. Vascular abnormality was defined as the presence of arteriolar hyalinosis and arteriosclerosis. The immunofluorescence study was performed using the classic direct technique with antibodies against 8 proteins (IgG, IgM, IgA, C3, C1q, fibrinogen, kappa and lambda chains). The results were reported semi-quantitatively as negative (0), trace (0.5) and 1–3 positive (1-3). The findings by electron microscopy were descripted as followings; presence of electron dense deposits in the area of the mesangium, subendothelium, and subepithelium; and severity of foot process effacement of the podocytes, which were reported as none, focal (mild, moderate, moderate to severe, severe), or diffuse.” (8p)

Lines 140-141
severity of foot process effacement of the podocytes, which were reported as none, focal (mild, moderate, moderate to severe, severe), or diffuse.

Comment
I believe that if there is only mild to moderate foot process effacement it is possible that the diagnosis of MCD is incorrect

Thank you for your comment.

As you said, it should be not descriptive of MCD. We just wanted to descript reviewed pathologic findings in detail. To clarify our intends, we fixed sentences as below. (8p in the manuscript). And we also abbreviated table 2 and added details of pathologic findings in supplementary table 1.

“As we had previously described [10], pathologic diagnosis contains following contents. Glomerular lesions such as global sclerosis, segmental sclerosis, glomerular ischemic change, and crescentic changes were reported as a proportion of the total number of glomeruli in an evaluated specimen. A semi-quantitative assessment of changes in mesangial cellularity, mesangial matrix, tubular atrophy, and interstitial inflammation and fibrosis was performed, with the results classified as normal, mild, moderate, moderate to severe, and severe. Vascular abnormality was defined as the presence of arteriolar hyalinosis and arteriosclerosis. The immunofluorescence study was performed using the classic direct technique with antibodies against 8 proteins (IgG, IgM, IgA, C3, C1q, fibrinogen, kappa and lambda chains). The results were reported semi-quantitatively as negative (0), trace (0.5) and 1–3 positive (1-3). The findings by electron microscopy were descripted as followings; presence of electron dense deposits in the area of the mesangium, subendothelium, and subepithelium; and severity of foot process effacement of the podocytes, which were reported as none, focal (mild, moderate, moderate to severe, severe), or diffuse.” (8p in the manuscript)

Lines 187-188
The patients were divided into Non-NS (n=20, 25.3%) and NS-groups (n=59, 74.7%) according to the presence of proteinuria ≥ 3g/g on the day of biopsy

Comment

Table 2 shows that 4 out of 20 patients in the non-NS group had a max UPCR ≥3.00 g/g cr before biopsy. However, on the day of the biopsy all patients on the non-NS group already had a UPCR of ≤ 3.00 g/g cr. This means that in some patients with MCD and nephrotic proteinuria the amount of proteinuria was already spontaneously decreasing without therapy.
I suggest to classify these patients in the nephrotic syndrome group as opposed to the non-NS group.
Maybe you can refer to both the max UPCR before or at the time of kidney biopsy to stratify patients in each one the groups

We are thankful for your recommendation.

Comparing two groups divided by maximal amount of proteinuria during 6 months before renal biopsy was done. It showed similar results with previous comparison with the amount of proteinuria on the day of renal biopsy. This result was added to the supplementary materials (S1 Table, S2 Table) and descripted in the manuscript as below.

“4 patients out of 20 patients who belonged to Non-NS group had a max UPCR ≥ 3g/g creatinine before biopsy. When dividing patients according to the maximal amount of proteinuria 6 months before biopsy by 3g/g creatinine, 16 patients had < 3 g/g creatinine of proteinuria and the other 63 patients had over 3g/g creatinine of proteinuria. When comparing groups according to a max UPCR before biopsy, the two groups showed high UPCR, lower GFR, and higher proportion of AKI (S1 Table).” (12p in the manuscript)

” These trend of clinical outcome was not different when analyzing patient divided by the maximal amount of proteinuria during 6 months before biopsy (S4 Table).” (18p in the manuscript)

**Supplementary Table 1. Characteristics of patients with minimal change disease according to the highest amount of proteinuria during 6 months before renal biopsy**

| **Characteristic** | **Completeness** | **Non-NS2** | **NS2** | **p-value** |
| --- | --- | --- | --- | --- |
|  | **of data (%)** | **(n=16)** | **(n=63)** |  |
| **Age (years)** | 100.0 | 52.4 ± 15.9 | 54.0 ± 20.0 | 0.729 |
| **Male (n, %)** | 100.0 | 7 (43.8) | 31 (49.2) | 0.696 |
| **DM (n, %)** | 100.0 | 5 (31.3) | 7 (11.1) | 0.045 |
| **Hypertension (n, %)** | 100.0 | 7 (43.8) | 35 (55.6) | 0.398 |
| **History of CHD (n, %)** | 100.0 | 0 (0.0%) | 4 (6.3%) | 0.301 |
| **Weight (kg)** | 100.0 | 63.9 ± 11.6 | 65.4 ± 12.8 | 0.644 |
| **SBP (mmHg)** | 100.0 | 118 ± 15 | 125 ± 18 | 0.109 |
| **DBP (mmHg)** | 100.0 | 70 ± 10 | 73 ± 12 | 0.232 |
| **Cholesterol (mg/dl)** | 93.7 | 255 ± 158 | 367 ± 115 | 0.016 |
| **Glucose (mg/dl)** | 100.0 | 129 ± 34 | 109 ± 29 | 0.046 |
| **Protein (g/dl)** | 92.4 | 5.8 ± 1.2 | 4.4 ± 0.8 | <0.001 |
| **Albumin (g/dl)** | 92.4 | 3.3 ± 1.0 | 2.2 ± 0.6 | <0.001 |
| **Hemoglobin (g/dl)** | 100.0 | 13.4 ± 1.9 | 13.5 ± 2.1 | 0.896 |
| **Creatinine (mg/dl)** | 100.0 | 0.97 ± 0.90 | 1.19 ± 0.80 | 0.368 |
| **Creatinine before biopsy (mg/dl)** | 100.0 | 0.68 ± 0.22 | 0.66 ± 0.24 | 0.728 |
| **GFR (ml/min/1.73 m2)** | 100.0 | 96 ± 29 | 77 ± 35 | 0.041 |
| **AKI at biopsy (n, %)** | 100.0 | 1 (6.3) | 35 (55.6) | <0.001 |
| **Stage 1 (n, %)** | 100.0 | 0 (0.0) | 14 (40.0) | 0.005 |
| **Stage 2 (n, %)** | 100.0 | 0 (0.0) | 9 (25.7) |  |
| **Stage 3 (n, %)** | 100.0 | 1 (100.0) | 12 (34.2) |  |
| **AKI requiring dialysis (n, %)** | 100.0 | 0 (0.0) | 5 (7.9) | 0.244 |
| **Max. UPCR before biopsy (g/g cr)** | 100.0 | 1.80 ± 0.87 | 10.88 ± 6.19 | 0.009 |
| **<0.30 g/g cr (n, %)** | 100.0 | 0 (0.0) | 0 (0.0) | <0.001 |
| **0.30-2.99 g/g cr (n, %)** | 100.0 | 16 (100.0) | 0 (0.0) |  |
| **≥3.00 g/g cr (n, %)** | 100.0 | 0 (0.0) | 63 (100.0) |  |
| **UPCR at biopsy (g/g cr)** | 100.0 | 1.58 ± 0.98 | 9.55 ± 6.46 | <0.001 |
| **<0.30 g/g cr (n, %)** | 100.0 | 2 (12.5) | 1 (1.6) | <0.001 |
| **0.30-2.99 g/g cr (n, %)** | 100.0 | 14 (87.5) | 3 (4.8) |  |
| **≥3.00 g/g cr (n, %)** | 100.0 | 0 (0.0) | 59 (93.6) |  |

**Supplementary Table 4. Outcomes of minimal change disease according to the highest amount of proteinuria during 6 months before renal biopsy**

| **Outcomes** | **Completeness** | **Non-NS2** | **NS2** | **p-value** |
| --- | --- | --- | --- | --- |
|  | **of data (%)** | **(n=16)** | **(n=63)** |  |
| **FU duration after biopsy (months)** | 100.0 | 60.4 ± 56.1 | 72.9 ± 48.2 | 0.424 |
| **Number of laboratory tests during follow-up period (/month)** | 100.0 | 0.42 ± 0.25 | 0.70 ± 0.79 | 0.021 |
| **Min. UPCR after biopsy (g/g cr)** | 100.0 | 0.21 ± 0.25 | 0.13 ± 0.26 | 0.279 |
| **<0.30 g/g cr (n, %)** | 100.0 | 12 (75.0) | 59 (93.7) | 0.027 |
| **0.30-2.99 g/g cr (n, %)** | 100.0 | 4 (25.0) | 4 (6.3) |  |
| **≥3.00 g/g cr (n, %)** | 100.0 | 0 (0.0) | 0 (0.0) |  |
| **Max. UPCR after biopsy (g/g cr)** | 100.0 | 3.00 ± 4.10 | 8.01 ± 9.48 | 0.043 |
| **<0.30 g/g cr (n, %)** | 100.0 | 3 (18.8) | 11 (17.5) | 0.122 |
| **0.30-2.99 g/g cr (n, %)** | 100.0 | 8 (50.0) | 16 (25.4) |  |
| **≥3.00 g/g cr (n, %)** | 100.0 | 5 (31.3) | 36 (57.1) |  |
| **UPCR at last visit (g/g cr)** | 100.0 | 0.91 ± 1.74 | 1.27 ± 4.47 | 0.614 |
| **<0.30 g/g cr (n, %)** | 100.0 | 10 (62.5) | 48 (76.2) | 0.279 |
| **0.30-2.99 g/g cr (n, %)** | 100.0 | 5 (31.3) | 9 (14.3) |  |
| **≥3.00 g/g cr (n, %)** | 100.0 | 1 (6.3) | 6 (9.5) |  |
| **Creatinine at last visit (mg/dl)** | 100.0 | 1.02 ± 0.68 | 1.01 ± 0.65 | 0.954 |
| **GFR at last visit (ml/min/1.73 m2)** | 100.0 | 87 ± 29 | 85 ± 30 | 0.756 |
| **Treatment after renal biopsy** |  |  |  |  |
| **RAS blocker (n, %)** | 100.0 | 10 (62.5) | 33 (52.4) | 0.468 |
| **Anti-hypertensive medication (n, %)** | 100.0 | 11 (68.8) | 43 (68.3) | 0.970 |
| **Anti-diabetic medication (n, %)** | 100.0 | 5 (31.3) | 24 (38.1) | 0.612 |
| **Treatment for induction of the first CR** |  |  |  |  |
| **Immunosuppression (n, %)** | 100.0 | 6 (37.5) | 56 (88.9) | <0.001 |
| **Steroid only (n, %)** | 100.0 | 5 (31.3) | 23 (36.5) |  |
| **Steroid and calcineurin inhibitor (n, %)** | 100.0 | 0 (0.0) | 28 (44.4) |  |
| **Steroid and cyclophosphamide (n, %)** | 100.0 | 1 (6.3) | 5 (7.9) |  |
| **Highest dose of prednisolone (mg/kg/day)** | 100.0 | 0.97 ± 0.05 | 0.88 ± 0.17 | 0.222 |
| **Total dose of prednisolone until CR or 1st relapse (mg)** | 100.0 | 4477 ± 1468 | 7063 ± 3518 | 0.081 |
| **Outcomes** |  |  |  |  |
| **The first CR (n, %) among patients with UPCR ≥3.00 g/g cr (n, %)** | 100.0 | 12/16 (75.0) | 58/63 (92.1) | 1.000 |
| **The first relapse among patients with UPCR <0.30 g/g cr (n, %)** | 100.0 | 5/12 (41.7) | 28/58 (48.3) | 0.676 |
| **SD at the first relapse (n, %)** | 100.0 | 1/16 (6.3) | 10/63 (15.9) | 0.122 |
| **Number of relapses after biopsy (/year)** | 100.0 | 0.23 ± 0.33 | 0.25 ± 0.41 | 0.830 |
| **CR at last visit (n, %)** | 100.0 | 13 (65.0) | 45 (76.3) | 0.384 |
| **Slope of GFR (ml/min/1.73 m2/year)** | 100.0 | (-)4.67 ± 8.97 | 3.13 ± 12.9 | 0.026 |
| **Renal events during follow-up (n, %)** | 100.0 | 2 (12.5) | 17 (27.0) | 0.226 |
| **ESRD during follow-up (n, %)** | 100.0 | 1 (6.3) | 9 (14.3) | 0.388 |
| **Death during follow-up (n, %)** | 100.0 | 0 (0.0) | 3 (4.8) | 0.374 |

Min UPCR after biopsy: the lowest value of UPCR during the follow-up period starting 1 month after biopsy, Max UPCR after biopsy: the highest value of UPCR during the follow-up period starting 1 month after biopsy, FU duration after biopsy: follow-up duration between renal biopsy and the last test of UPCR, RAS: renin-angiotensin-system, CR: complete remission of proteinuria <0.3 g/g creatinine, relapse: UPCR >3.0 g/g creatinine after achieving CR of UPCR, SD: presence of steroid dependency in a patient where relapse of UPCR occurred during steroid tapering or within 2 weeks after cessation of steroids, renal event: any decrease of GFR of more than 50% during a follow-up visit compared to that at renal biopsy, GFR <15 ml/min/1.73 m^2^, or development of ESRD, The first CR (n, %) among patients with UPCR >3.00 g/g cr: In the Non-NS group, patients who had increased proteinuria of >3.00 g/g cr during observation period. The first relapse among patients with UPCR <0.30 g/g cr: In the Non-NS group, patients who had UPCR of <0.30 g/g cr at any time of period.

Lines 198-199
Serum levels of protein and albumin were lower in the NS group, as were the levels of total cholesterol (p<0.001, table 2)

Comment

Table 2 shoes that cholesterol levels were higher in the NS group which is the opposite of what is mentioned on the text. As expected, patients with nephrotic syndrome had higher cholesterol levels

I really appreciate your comment. Description in lines you mentioned should be corrected as below. (12p in the manuscript)

“. Serum levels of protein and albumin were lower in the NS group, when the level of total cholesterol was higher (p <0.001, Table 2).” (12p in the manuscript)

Lines 207-208
Interestingly, two patients in the NS group and one in the Non-NS group showed no podocyte effacement.

Comment
Please clarify this. The definition for MCD proposed mentions that patients should have focal or diffuse foot process effacement. If these patients did not have podocyte effacement, how were they diagnosed with MCD?

Thank you for your comment. The sentences refer to 2 patients who are suspected to have had MCD change before biopsy. As we mentioned in discussion, there are controversies on the correlation between the amount of proteinuria and the severity of podocyte effacement. Although we showed higher relative risk on the podocyte effacement and the amount of proteinuria, it did not consistent .

Lines 209-210 Table 2

Comment
Table 2 shows on immunofluorescence that there is staining for IgG, IgA, C3 or C1q in multiple patients.
Classic MCD does not have immunoglobulin or complement deposits. There may be variants of MCD (eg, IgM nephropathy or C1q nephropathy). But it is unclear if this represent separate conditions. Moreover, the presence of Ig M deposits in a patient with MCD has been associated with a poorer prognosis and a lower response to glucocorticoids compared to patients with MCD without deposits
Moreover, the proportion of patients with diffuse process effacement in the non-NS group is low (40%) which in my opinion raises questions about whether they had the correct diagnosis.

Thank you for your recommendation.

As you suggested, it was our limitation. We would describe this in discussion as a limitation as below.

“There are some limitations to this study. This was retrospective study, and the confounders were not strictly controlled. Thus, we only included patients without previous immunosuppressive treatment. In addition, the use of strict inclusion criteria meant that many patients were excluded from the study. As a result, the number of patients included was small. Second, only adults were included in this study, despite MCD being prevalent in children. However, the clinical course of MCD differs in various aspects between adults and children. Thus, it was meaningful to specifically describe the prognosis of MCD in adults. In the same sense, further studies that consider the initial amount of proteinuria are required for patients of all ages. Third, the pathologic findings in some patients were away from classic MCD in our study. Findings were EM findings such as less than 50% of podocyte effacement, and IM findings such as mild deposits. This might because MCD sometimes manifested atypically. Although our study suggested that there was no definite correlation with pathologic severity and the clinical features such as the amount of proteinuria, we still need further findings to prevail this.”(25p)
