# Supplementary material for: Outcomes of minimal change disease without nephrotic range proteinuria
Source: PLoS One. 2023 Aug 17;18(8):e0289870. doi: 10.1371/journal.pone.0289870 (PMC10434851; doi:10.1371/journal.pone.0289870)
Supplement: Attachment — Submitted filename: AnswerForReviewers.docx [file pone.0289870.s008.docx]

**1. please add how do you identify if the biopsy findings are related to MCD vs. unsampled FSGS? As we are not able to rule out the possibility of unsampled FSGS, a comment on this limitation should be addressed.**

Thank you for comments. The samples included in this study showed more than 10 glomeruli to maintain sampling adequacy. To Differentiate between MCD and unsampled FSGS, tubulointerstitial damage would have been great to be reported. So, we added this limitation to discussion section.

(Reference.

Rosenberg AZ, Kopp JB. Focal Segmental Glomerulosclerosis [published correction appears in Clin J Am Soc Nephrol. 2018 Dec 7;13(12):1889]. *Clin J Am Soc Nephrol*. 2017;12(3):502-517. Doi:10.2215/CJN.05960616)

So, we added some sentences as follows. Some sentences are red colored that are major revision.

“Third, the pathologic findings in some patients were away from classic MCD in our study. Findings were EM findings such as less than 50% of podocyte effacement, and IM findings such as mild deposits. This might because MCD sometimes manifested atypically. Although our study suggested that there was no definite correlation with pathologic severity and the clinical features such as the amount of proteinuria, we still need further findings to prevail this. “

🡪

“Third, the pathologic findings in some patients were away from classic MCD in our study. Findings were EM findings, such as < 50% of podocyte effacement, and IM findings, such as mild deposits. Tubulointerstitial damage to differentiate between MCD and unsampled FSGS was not thoroughly investigated. This might be because MCD is sometimes manifested atypically. However, we tried sampling adequate glomeruli to maintain sampling adequacy. Our study suggested no definite correlation between pathologic severity and clinical features such as the amount of proteinuria; however, we still need further findings to prevail this. “(21p)

**2. Clarify in the manuscript that patients in the non-NS group did not receive any other immunosuppression therapy other than the 8 patients who received steroids.**

Thank you for recommendation. Sentence about your comment was revised as follows. Some sentences are red colored that are major revision.

“The 8 patients received steroid treatment.”

- “These eight patients received steroid treatment without other immunosuppression therapy.” (13p)

**3. For the patients that received other immunosuppressive therapy in the NS group, please clarify the immunosuppression used- specifically, did any of them got rituximab?**

Thank you for your comments. Patients in our study did not use rituximab in study period. Other immunosuppressive therapies such as calcineurin inhibitors and cyclosporine were used in some patients. I descripted more details as below. Some sentences are red colored that are major revision.

“Steroids were administered to 93.2% of patients (55/59) in the NS group. The proteinuria spontaneously improved to a UPCR <0.3 g/g creatine in 4 patients. Among these, 2 patients remained in remission, 1 experienced an increase to UPCR 0.30–0.29 g/g creatinine, and 1 experienced relapse of proteinuria. The maximum dose of prednisolone administered to the 55 patients in NS group was 0.88 ± 0.17 (range: 0.4–1.2) mg/kg/day. Remission of proteinuria was achieved in 51 patients. Eventually, 55 out of 59 patients in NS group achieved a first remission of proteinuria, 4 without treatment and 51 with steroid treatment. Of these, 25 patients ultimately experienced a relapse.”

🡪” Steroids were administered to 93.2% of patients (55/59) in the NS group. The maximum dose of prednisolone administered to the 55 patients in the NS group was 0.88 ± 0.17 (range: 0.4–1.2) mg/kg/day. The proteinuria spontaneously improved to a UPCR <0.3 g/g creatine in four patients. Among these, two patients remained in remission; one increased in UPCR to 0.30-0.29 g/g creatinine, and one experienced a relapse of proteinuria. Thirty-seven out of 55 patients received steroids only, while 14 patients received steroids and calcineurin inhibitors, and four received steroids and cyclophosphamide. Among them, remission of proteinuria was achieved in 51 patients. Eventually, 55 out of 59 patients in the NS group achieved the first remission of proteinuria. Four (6.8%) achieved the first remission without any immunosuppressive treatment. Among 51 patients who used steroids, 34 patients (61.8%) used steroids only, while 13 patients (23.6%) with calcineurin inhibitors, and four patients (7.3%) with cyclophosphamide.” (13p)

**4. What is the authors' input in the possibility that some of the subjects in the non-NS group may be just at a different period of the spectrum of MCD, and they may progress to develop NS afterwards?**

I sincerely appreciate for your recommendation. This paper was authored with the intention of presenting data that suggests the possibility of progression from a subclinical state to nephrotic syndrome, thereby highlighting the potential for manifestation even in cases that do not initially exhibit clinical symptoms.

**5. Clarify how many achieved spontaneous remission in both groups, and the period of time in which patients achieved spontaneous remission after diagnosis.**

16 out of 20 patients (80%) in non-NS group achieved the first CR spontaneously, when 4 out of 59 patients (6.8%) in NS-group did. All 4 patients who had a remission spontaneously were belong to the NS-group. The average period of the first relapse to remission was 33.6 months (±64.31 months). 1 patient among these showed relatively long duration from biopsy day to the day of remission, which was 130.0 months. The amount of proteinuria in this patient decreased, not enough to achieve remission for a while. Except this patient, other three patients showed average 1.42 months (±2.24 months). Meanwhile, others who experienced remission in non-NS group received steroid only.
