# Supplementary material for: Outcomes of minimal change disease without nephrotic range proteinuria
Source: PLoS One. 2023 Aug 17;18(8):e0289870. doi: 10.1371/journal.pone.0289870 (PMC10434851; doi:10.1371/journal.pone.0289870)
Supplement: Attachment — Submitted filename: AnswerForReviewers.docx [file pone.0289870.s009.docx]

**1. In your last sentence of introduction, where you are expressing the objective of your study, you mention: "we compared the clinicopathologic features and outcomes between MCD patients with and without heavy proteinuria for approximately 5 years." However, in your conclusion paragraph your wording is different than your objective: "In conclusion, there was no difference in the prognosis of MCD between patients with and without spontaneous remission. Patients who experience spontaneous remission of MCD should be monitored as closely as those with nephrotic MCD without spontaneous remission." Please unify your wording to match between your objective and your conclusion. Because the current conclusion does not match the objective of the study.**

Thank you for comments. The sentences in conclusion were corrected into more concise sentences to deliver the author’s point as below.

“In conclusion, there was no overall clinicopathological difference in the prognosis of MCD between patients with and without heavy proteinuria. Therefore, patients who show non-nephrotic proteinuria as an initial manifestation should be monitored as closely as those with nephrotic MCD without spontaneous remission.” (23p)
