# Supplementary material for: Outcomes of minimal change disease without nephrotic range proteinuria
Source: PLoS One. 2023 Aug 17;18(8):e0289870. doi: 10.1371/journal.pone.0289870 (PMC10434851; doi:10.1371/journal.pone.0289870)
Supplement: Attachment — Submitted filename: AnswerForReviewers.docx [file pone.0289870.s010.docx]

1. **Consider switching the Title to "****Outcomes of Minimal Change Disease without Nephrotic Range Proteinuria", which is more representative of the manuscript goals. I hope authors agree.**

Thank you for the editors’ recommendation. We agree to change the title as you suggested. Short title was also changed to “Minimal Change Disease with or without Nephrotic Range Proteinuria”. Thank you.

**2. The reference 6 provided by the authors states that all the asymptomatic subjects either had proteinuria of 2g or low serum albumin of 2 (this last one fulfills criteria for NS) but authors describe it in their manuscript as MCD with no NS which is not true, please edit line 63.**

I sincerely thank you for your recommendation. After reviewing the reference, we found that it was not appropriate to deliver our opinion in introduction due to misinterpretation. We changed the sentence as follows. Reference numbers were changed according to the deletion of the reference in Introduction section.

“In adults, MCD exhibits a relatively large level of proteinuria compared with other primary glomerulonephritis. However, the long-term prognosis of adult MCD without nephrotic syndrome as an initial manifestation is unclear. There are some studies on the prognosis of MCD without nephrotic syndrome in children [6]. Patients with an initial presentation of proteinuria < 2 g/day/m^2^ did not experience relapses within 1 year, while those with nephrotic syndrome did. Children with lower levels of proteinuria were also more sensitive to steroid treatment. However, the prognosis of MCD with typical pathologic findings needs to be defined in terms of clinical characteristics, response rates, and relapse rates in patients with and without nephrotic syndrome. Therefore, we compared the clinicopathologic features and outcomes between MCD patients with and without heavy proteinuria for approximately 5 years.”

- “In adults, MCD exhibits a relatively large level of proteinuria compared with other primary glomerulonephritis. Until this study, most studies have focused on heavy proteinuria diagnosed with MCD. Therefore, the long-term prognosis of adult MCD without nephrotic syndrome as an initial manifestation is unclear. To fully understand MCD, the prognosis of MCD with typical pathologic findings needs to be defined in terms of clinical characteristics, response rates, and relapse rates in patients with and without nephrotic syndrome. In this study, we compared the clinicopathologic features and outcomes between MCD patients with and without heavy proteinuria for approximately 5 years.” (line 61–67, 4p)

**3. In line 134, please clarify if all the subjects had EM performed, or what percentage of the study population had EM performed. Consider that if not all subjects had EM, then the diagnosis of MCD is not possible.**

Thank you for recommendation. We added the sentence as follows.

Findings on EM were described as follows; the presence of electron-dense deposits in the area of the mesangium, subendothelium, and subepithelium; and severity of foot process effacement of the podocytes, which were reported as none, focal (mild, moderate, moderate to severe, severe), or diffuse.

- “After EM analysis was done in all biopsy samples, findings on EM were described as follows; the presence of electron-dense deposits in the area of the mesangium, subendothelium, and subepithelium; and severity of foot process effacement of the podocytes, which were reported as none, focal (mild, moderate, moderate to severe, severe), or diffuse.” (line 131–135, 8p)
